# The potential of single-cell profiling in plants

**DOI:** 10.1186/s13059-016-0931-2

**Published:** 2016-04-05

**Authors:** Idan Efroni, Kenneth D. Birnbaum

**Affiliations:** The Center for Genomics and Systems Biology, Department of Biology, New York University, New York, NY 10003 USA; Present address: The Robert H. Smith Institute of Plant Sciences and Genetics in Agriculture, The Hebrew University, Rehovot, 76100 Israel

## Abstract

Single-cell transcriptomics has been employed in a growing number of animal studies, but the technique has yet to be widely used in plants. Nonetheless, early studies indicate that single-cell RNA-seq protocols developed for animal cells produce informative datasets in plants. We argue that single-cell transcriptomics has the potential to provide a new perspective on plant problems, such as the nature of the stem cells or initials, the plasticity of plant cells, and the extent of localized cellular responses to environmental inputs. Single-cell experimental outputs require different analytical approaches compared with pooled cell profiles and new tools tailored to single-cell assays are being developed. Here, we highlight promising new single-cell profiling approaches, their limitations as applied to plants, and their potential to address fundamental questions in plant biology.

## Introduction

Many of the distinguishing features of plants are attributable to the functions of highly specialized cells. Transcriptomic analysis of these specialized cells has significantly advanced our understanding of key events in plant development, such as tissue specification in the root [1, 2] and shoot [3] or stomatal maturation [4]. Tissue-specific profiling has also shown that environmental conditions lead to dramatically different responses in various cell types [5, 6]. These advances rely on fluorescent protein markers that have enabled the tracking and isolation of cell populations of particular identity.

However, the markers used to profile cells were largely chosen for their ability to represent anatomical features and many fundamental questions would benefit from an unbiased view of cellular organization. For example, physiology can call for cellular specialization where anatomy does not. In addition, the full extent of cellular variation in response to biotic and abiotic stresses is not well characterized, as different cells clearly respond differently, for example, to pathogen attacks [7, 8]. In some cases, we simply lack good markers for crucial cell populations. For example, no single reporter uniquely marks the root initials and the signals that regulate stem cell activity remain poorly understood [9]. Furthermore, while development is a dynamic process, most of the current cell-type profiles confound multiple developmental stages. A continuous progression of cell states from birth to differentiation is required to reveal how cells regulate their maturation [10].

In this *Opinion*, we focus on how single-cell RNA-seq can be used to dissect plant tissue organization, developmental dynamics, and physiological responses (Table 1). Based on early studies, single-cell RNA-seq protocols developed for animal systems have produced high-quality profiles in plant cells [11, 12], as we detail below. We first address cell isolation issues that are specific to plants. For mRNA amplification and library preparation methods common to plants and animals, we refer the readers to a recent comprehensive review [13]. We then focus our discussion on three analytical topics that are of central importance in mining single-cell data in plant studies—discriminating technical versus biological noise, detecting distinct cell types, and ordering developmental trajectories.

**Table 1:** Questions in plant biology to which single cell profiling could be applied: analytical problems and algorithmic solutions

Biological problem or plant-specific question	Analytical problems for single-cell data	Potential approaches
Distinguish genes that show true biological variation	Significant technical noise is present	Hypothesis testing based on identification of variation that exceeds empirical estimations of technical noise [11]
What genes vary among physiologically distinct cells of seemingly homogenous tissues?	Profiles have no replicates and exhibit zero-biased expression distribution, so traditional statistical methods are inappropriate	
		Model-driven deconvolution of biological variation using estimations of technical noise [20]
Identify transcriptional signature of rare cell types	Linear dimensionality reduction can obscure close relationships and produce misleading clusters	Non-linear t-SNE to minimize joint probability distribution distance and draw similar cells together [29]
What is the transcriptional profile of root initials?	Clustering methods might miss small sets of cells	backSPIN to impose an order and partition data [31, 32]
Find subsets of cells with a unique environmental response	Separation of a continuous cell expression space into types is subjective	RaceID to identify new cell types by detecting a significant number of biological gene outliers [30]
What is the early response of pathogen-susceptible vs. pathogen-resistant cells of the leaf epidermis?		
Assemble dissociated cells into a developmental sequence	Missing data-points exist owing to false negatives and misleading false positives	De novo trajectory reconstruction to order cells using Monocle [39] or diffusion-like dynamics [40]
What is the ordered profile of specific cell types from initial to differentiated cells?	Variation in individual plants can create artificial groupings	Seurat to map cells using a priori data and imputation of missing data-points [41]
		ICI to map cells to known reference types using many markers [12, 38]
Follow identity transitions during wound repair or in vitro regeneration	Detecting transitional and multiple identities must be robust in single-cell data with many false positives and false negatives	ICI to classify cells using a priori knowledge of identity markers for detecting mixed or diminished cell identity [12, 38]
Do plant cells follow a course of de- or trans- differentiation during regeneration?		

*ICI* index of cell identity, *t-SNE* t-distributed stochastic neighbor embedding

## Isolation of single cells from plants

Plant cells are immobilized in a rigid cell wall matrix that must be removed or penetrated. External cells are more accessible and early studies at the single-cell level used microcapillaries to manually extract their protoplasm (e.g., [14]). However, in order to profile a large numbers of cells or cells from internal tissue, the most feasible method is enzymatic cell wall digestion. This is routinely achieved by incubating plant tissues in cellulases and other cell-wall-degrading enzymes for as little as one hour, releasing individual protoplasts into solution [15, 16].

In order to isolate fluorescently labeled cells, two recent plant studies have used glass micropipettes to aspirate single fluorescently labeled cells under a stereomicroscope with epifluorescence [11, 12]. However, this method is very labor intensive and is only practical for profiling of, at the most, a few dozen cells. For higher-throughput studies, fluorescence-activated cell sorting (FACS) is currently the most commonly used method for single-cell isolation. FACS can distribute individual cells into 96- or 384-well plates and we do not anticipate major problems with this technique in plants, as pooled sorting of plant protoplasts works well. Recently, higher-throughput microfluidics-based methods that can process tens- to hundreds-of-thousands of cells were developed for animal cells [17, 18]. These methods are promising for widespread use, although they have not yet been tested on plant cells and are not currently commercially available.

The cell walls of some plant tissues are particularly recalcitrant to cell wall digestion, including more-mature tissues with secondary cell walls. An approach that could address this problem is the isolation of nuclei from internal tissue, for example, by tissue chopping [19]. The profiling of pooled nuclei from specific cell types has been performed in plants and appears to reflect known cell-specific expression [20]. In principle, techniques for RNA-seq from single nuclei developed in animals [21] could be applied to plants with little or no modification. However, as nuclei were shown to contain only ~10 % of the cellular RNA [20], one open technical issue is how much the lower RNA yield would affect technical sampling noise (see below).

## Biological versus technical variability

**Fig. 1:**
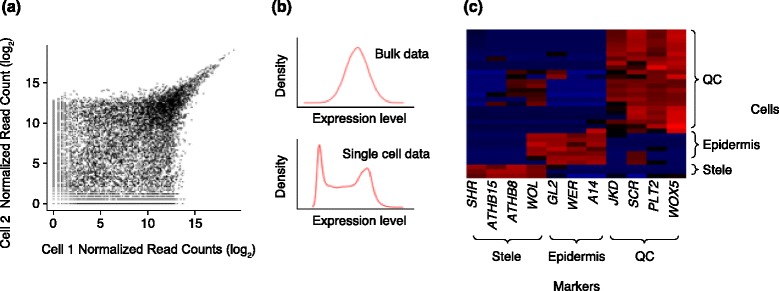
Single-cell transcriptomic profiles in plants. **a** The technical noise profile between two single cells of the same cell type, showing high dispersion for transcripts expressed at a low level. The *axes* are read-counts representing gene expression levels on a log2 scale. As most genes are expected to be expressed at similar levels, the two axes evaluate replication and show that, at these scales, genes expressed at higher levels show the potential to distinguish biological from technical noise. **b** (*upper*) The expression distribution of a gene among pooled samples typically shows a peak frequency on a positive expression value. (*lower*) Gene expression among single-cell samples typically shows a peak frequency at zero, with a subset of cells showing a second peak of positive read counts in a subset of samples. Density represents the frequency of cells showing a given expression level (read count). **c** Several gold-standard markers in single-cell profiles of cells with known tissue origins. These functional markers are expressed at higher levels (e.g., more replicable expression in **a** and non-zero expression in **b** (*lower*). In these real samples collected from plant cells, markers for the quiescent center (*QC*), stele, and epidermis all show detectable expression in target cells and are largely absent in non-target cells, with some false-positive and false-negative expression

One of the goals of transcriptional profiling is the identification of differentially expressed genes between samples. Traditional statistical models rely on the use of replicates to identify differentially expressed genes. In the typical experimental design of single cell transcriptomics, however, all cells are considered independent biological samples, creating the need for methods tailored to single-cell outputs. The lack of true replicates is of special concern as low initial mRNA molecule number produces considerable technical noise. This is apparent by the high dispersion of gene expression, especially at low levels, when comparing two similar cells (Fig. 1a) [11, 22–25]. The technical variability stems mainly from the inefficient process of cDNA synthesis [25], resulting in sequencing libraries that represent only about 10 % of the original mRNA population in the cell [23]. The sampling process introduces Poisson-distributed noise that dominates low expression levels (Fig. 1a). In particular, transcripts with low copy number are often omitted, producing zero biased expression-level distributions, which are greatly different from the positive mean tendencies of pooled cells (Fig. 1b). The zero-based property will affect background null distributions for statistical analysis. Despite the technical noise, however, many functional cell-specific markers, including those in plants, appear to be expressed at high enough levels to show robust expression, with relatively low rates of observed false negatives or false positives (Fig. 1c) [12].

Two general approaches have been used to estimate technical noise and deconvolute true biological variability in gene expression among single cells. Brennecke and colleagues [11] used both plant and animal single-cell profiles to model technical noise based on spike-in RNA, which they use to produce a *p* value for each gene that addresses the hypothesis that the biological variability of a gene in a population of cells exceeds the predicted technical noise [11]. In a different approach, Grün and co-workers [23] modeled gene expression distributions, accounting for both sampling noise and global cell-to-cell variability. This group used spike-in data to fit a formal model of noise based on commonly used distributions [23]. This method could also be used on plant single-cell profiles as technical noise has characteristics identical to those of animal cells (e.g., Fig. 1a) [23]. One lesson learned from these early studies is that a denser RNA spike-in, such as total RNA from a distantly related organism [11], can provide a more accurate noise estimation than the standard set of 92 spike ins [23].

Application of such methods to isolated root cells has led to the identification of many genes whose expression varied among single cells, even from seemingly uniform tissues [11]. However, in order to understand the biological meaning of such variability, the resulting gene list has to be cross-referenced with other databases. *Arabidopsis* has rich gene expression resources that can be used to identify markers for biological processes. For example, a repository of tissue-specific gene expression data was used to translate changes in gene expression to changes in cell identity during plant regeneration [12]. Analysis of *cis*-regulatory data is also a useful tool in identification of common modules and potential regulators, as evidenced by the identification of novel muscle differentiation regulators in human cells [26]. However, profiling of DNAse-hypersensitivity data in plants is currently sparse (but see [27]).

## Discovery of unique cell states

While anatomy has been the traditional guide to cell-type classification, single-cell transcriptomics can, in principle, provide an unbiased approach to identify cell types or subtypes. This could be applied, for example, to sampling meristematic cells in search of a stem cell signature or cells of an infected leaf in order to detect differential cellular responses to pathogen attacks.

One common approach to cellular classification is mapping cells with high-dimensional transcriptional readouts in a low-dimensional space to identify coherent clusters. The most commonly used visualization technique for this approach is principal components analysis (PCA) [28]. Applied to cell grouping, the technique generates a cell-by-cell correlation matrix and then extracts axes, in order of explained variance, that capture gene expression patterns that best separate cell states. Another technique for dimension reduction—multi-dimensional scaling (MDS) [29]—finds a low-dimension (typically two) projection that will preserve as much as possible the distance between cells in the original high-dimension space. Several recent animal studies have used PCA or MDS followed by gene discovery [30, 31], for example, to identify new markers for cancer subtypes in glioblastoma [30].

Both of these dimensionality-reduction techniques use linear metrics, which can have the undesirable quality of spreading apart relatively similar cells in the transformation to lower dimensions [32]. We have observed, for example, that single-cell profiles from highly localized plant quiescent center (QC) cells are relatively dispersed in the first two axes of a PCA [12]. A non-linear dimensionality-reduction technique called t-distributed stochastic neighbor embedding (t-SNE [32]) has been used extensively in single-cell studies [17, 33, 34]. t-SNE converts gene expression differences between any two cells to a conditional probability that gene *x* is the nearest neighbor of gene *y*. The program makes the transformation from multiple to two or three dimensions by minimizing the joint probability distributions from high- to low-dimensional space, allowing adjustments in the transformation that, for example, lead to greater attraction of similar cells. Considering the differential response to plant cell infection, all sampled cells might share the same identity, giving them a highly similar background expression. If similar cells are dispersed in a low-dimensional space, a divergent subgroup might be hard to distinguish. A tight grouping of the non-responsive subset (for example, using t-SNE) could help distinguish the responsive group.

The methods above typically rely on a subjective definition of a cluster or cell type by visual inspection of the low-dimensional cell space. In the example above, partitioning the responsive and non-responsive cell groups by eye could introduce the potential for bias. More objective approaches to clustering and partitioning cells have also been developed. For example, the “sorting points into neighborhoods” (SPIN) method has been used to create a global ordering of cells. The technique builds a cell-by-cell correlation matrix and orders cells to form a pattern of high correlations along a continuous diagonal in the matrix [35]. A mouse study used the approach on 3005 cells from the brain using SPIN to order cells and then find breakpoints that divided cells into highly correlated subgroups along the ordered matrix (backSPIN [34]). In plants, this technique could be used on cells that form a developmental trajectory that exhibit discrete states, such as phase changes. For example, backSPIN could be used to partition cells into the meristematic, elongation, and differentiation zones. While these methods provide a formal way to cluster cells, they still require subjective cutoffs. In addition, more-standard techniques for partitioning clusters, such as gap statistics, have also been used to identify single-cell clusters [33].

Another problem is that subpopulations become increasingly difficult to detect from neighboring populations when they are rare. This is likely to be the case for plant stem cells, which can represent a small proportion of cells marked by cell-identity reporters. Thus, distinguishing a potential unique stem cell signature distinct from the neighboring cells will be challenging. In principle, a cell should only be called unique if it displays true biological variation from nearby cell states that exceeds the expected technical noise. Using such an approach, Grün and colleagues [33] extended their technical noise-deconvolution approach (see above [23]) to cell-type identification. The method, called RaceID, groups cells into clusters and then identifies genes whose expression in given cells of the cluster exceeds the technical noise [33]. Cells that had a significant number of outlier genes were deemed a novel subtype. This approach or more-empirical approaches to modeling technical noise (e.g., [11]) and identifying marker transcripts could prove useful for distinguishing a small group of candidate stem cell states in the meristem. Nevertheless, statistical power to distinguish differential expression will obviously improve with greater numbers of cells. Empirically, we have found differential expression to agree well with gold-standard markers when at least five cells of a given type are identified, but this number will vary according to the experimental set-up.

In some cases, the differential response of a group of cells might be a given, but it is their similarity to known states that is the crucial question. For example, a plant cell can rapidly change its identity in response to local [36] or extensive injury [37–39]. Whether plant cells do this through dedifferentiation or transdifferentiation or through novel states is an open question [40]. Resolving such questions requires an accounting of known cell fates among regenerating cells. One approach to this problem is to use many markers of known cell states to ‘vote’ on the identity of a cell in question. Thus, the first task is to quantify the specificity of a comprehensive set of cell-type- and developmental-stage-specific markers (e.g., [41]). We have developed an information-based approach to identify markers from known tissue-specific profiles [12]. We then used these markers to quantify cell identity [“index of cell identity” (ICI)] over background noise. The large number of markers reduced batch effects, was robust to noise, and permitted the detection of mixed identity. The method was used to show a transient loss of vascular identity in regenerating roots [12]. Overall, ICI represents a highly “supervised” alternative to cell-state discovery.

## Constructing developmental trajectories

In the plant meristem, cells are often arranged in maturation gradients in which their spatial position often correlates with developmental stage. Single cell mRNA-seq analysis provides an opportunity to assemble these developmental trajectories in fine detail. During the process of tissue disassociation, however, knowledge of the original position of a cell is lost, requiring bioinformatic inference of the development stage of the cell.

**Fig. 2:**
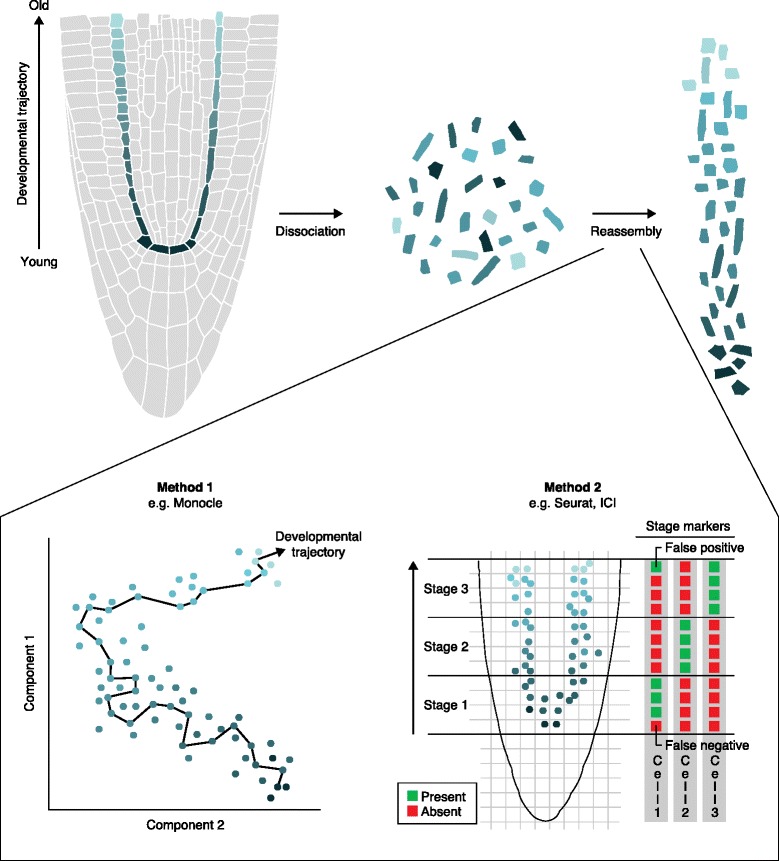
Hypothetical example showing the pseudo-time ordering of cells collected from the root meristem. (*upper*) The green-colored cells represent a reporter marking the endodermis and quiescent center (QC). The *color gradient* represents a continuum of cellular maturation from birth (at *bottom*) to differentiation (towards the *top*). Cells are dissociated and isolated using fluorescence-activated cell sorting (FACS), whereupon ordering information is lost. At the *right*, single-cell expression profiles are used to infer a pseudo-ordering as cells in an approximate sequence. (*lower*) Two general methods of pseudo-time ordering are shown. Method 1 is unsupervised, using dimensionality reduction to position cells in a hypothetical space and then imposing an optimal path that infers the developmental progression of cells (e.g., Monocle). Method 2 uses markers to place cells in a specific location or developmental zone, with specific approaches differing in the way they adapt to false negatives and false positives. Seurat infers the expression of missing “gold-standard” markers based on coexpressed genes. Index of cell identity (*ICI*) employs many markers that “vote” on cell localization, where misleading diagnostic markers from false positives and false negatives are overcome by a majority of true positives. (Schematic by Ramin Rahni)

One set of methods to reconstruct developmental trajectories from single cells relies on the assumption that neighboring stages show similarity of gene expression. One such method, Monocle, employs dimensionality reduction to plot cells on two axes and then charts a path through the cell space that represents a pseudo-time series using a minimal spanning tree (Fig. 2, Method 1) [26]. Alternatively, differentiation trajectories have been modeled using non-linear diffusion-like dynamics in a high-dimensional transcriptional space [42].

These approaches assume that developmental stage is the dominant signal in single-cell profiles. This might present a problem because plants are highly tuned to their microenvironment and even tightly controlled growth conditions will yield plant-to-plant differences in gene expression. Such plant-specific effects could create artifacts in a completely unguided de novo assembly of cell states, such as those above. Approaches that guide the assembly of cell states with some prior knowledge of cell states would help address this issue.

Seurat is a software package that uses a priori spatial information from the expression of a small number of known marker genes to deduce the position of cells in the original tissue [43]. In order to handle the technical sampling noise, Seurat uses clustering and machine-learning techniques to estimate, or “impute”, the expression level of what it infers to be missing markers (Fig. 2, Method 2). While the method was developed and customized for the analysis of the zebrafish embryo, a similar approach could be used for cells in plant meristems using a priori knowledge of the spatial expression of multiple markers, as is available for *Arabidopsis*, maize, rice, and a growing number of plant species. Alternatively, sets of genes that vote on the specific developmental stages of a cell can be used as a score for developmental stage, as could be implemented in the ICI approach [12]. Such a method could, for example, be used to place cells along a trajectory from stem cell to differentiated cell (Fig. 2, Method 2). One could envision using these protocols to describe a stem cell state and the discrete steps of differentiation that proceed it.

## Concluding remarks

Single-cell RNA-seq works as efficiently in plant cells as in animal cells. Noise profiles are well understood and an early set of analytical approaches is now capable of extracting information not previously possible in pooled samples. The biggest technical challenges to adapting single-cell protocols to plants will be dissociating cells from the appropriate tissues and obtaining high numbers of cells for high-throughput analysis. In addition, the technical noise associated with single-cell assays and the lack of true biological replicates pose a challenge in distinguishing differences in gene expression between single cells. The unsupervised grouping of cells before statistical analysis has been used to create de facto replicate samples, but researchers need to be cautious of batch effects that can dominate unsupervised clustering. Nonetheless, most of these problems are not unique to single-cell analysis and the ability to profile large numbers of cells can be leveraged to address noise and identify replicate cell states. Towards that end, multiple bioinformatic tools for the analysis of single-cell transcriptomes have been developed and successfully applied. Single-cell analysis of whole organs has the potential to identify highly localized responses to stress and environmental inputs, map developmental trajectories, and rapidly profile emerging models where specific fluorescent markers are not yet available (Table 1). Thus, in addition to the specific questions discussed herein, single-cell analysis holds the potential to generate datasets that could rapidly accelerate comparative developmental genomics at the cell level.

## Abbreviations

FACS: fluorescence-activated cell sorting
ICI: index of cell identity
MDS: multi-dimensional scaling
PCA: principal components analysis
QC: quiescent center
SPIN: sorting points into neighborhoods
t-SNE: t-distributed stochastic neighbor embedding
